# One (small) step towards precision nutrition by use of metabolomics

**DOI:** 10.1016/S2213-8587(17)30007-4

**Published:** 2017-01-13

**Authors:** Shilpa N Bhupathiraju, Frank B Hu

**Affiliations:** Department of Nutrition, Harvard T H Chan School of Public Health, and Channing Division of Network Medicine, Brigham and Women’s Hospital and Harvard Medical School, Boston, MA 02115, USA

Despite advances in nutritional epidemiological study design and analytical strategies, dietary assessment in free-living populations remains a major challenge.^1^ The habitual diet represents a complex set of exposures that are intercorrelated, and self-reported tools might suffer from random and systematic errors. Although several biomarkers of nutrient intake (eg, protein intake by urinary nitrogen, urinary sodium and potassium, and essential dietary fatty acids in plasma) exist, objective measurement of the overall dietary pattern has remained elusive. However, new omics technologies such as metabolomics might hold promise for the development of a robust and unbiased strategy for measuring diet. Metabolomics can measure the full profile of small-molecule metabolites in biofluids, thereby providing a comprehensive picture of a person’s overall dietary intake. Metabolite profiling accounts for intrinsic variability in metabolism by measuring downstream components or metabolic products of foods, and might therefore better accurately reflect true exposure than traditional methods that measure individual food intakes.^2^ Although some studies^3–7^ have identified metabolites associated with intake of certain foods, little research has been done in the identification of metabolite patterns that reflect the overall dietary pattern.

In *The Lancet Diabetes & Endocrinology*, Isabel Garcia- Perez and colleagues^8^ report the use of proton nuclear magnetic resonance (^1^H-NMR) spectroscopic profiling of urine to develop urinary metabolite patterns that can classify individuals on the basis of their overall diet. In a rigorously controlled crossover feeding study, 19 healthy participants consumed four defined diverse diets for 72 h each, separated by at least 5 days. The diets differed in compliance to the WHO healthy eating guidelines (decreased sugar, salt, and total fat consumption, and increased intake of whole grains, fruits, vegetables, and dietary fibre), with diet 1 being the most concordant with the guidelines and diet 4 the least concordant. Partial least squares discriminant analysis of 24 h urinary ^1^H-NMR spectral profiles showed systematic differences in metabolic profiles between diets 1 and 4 (Skillings-Mack test p=7·21 × 10^−9^), although some degree of overlap was seen in predicted scores across the four diets. Specifically, when comparing urinary metabolic profiles after consumption of diets 1 and 4, the investigators found 19 urinary metabolites to be present in higher concentrations after consumption of diet 1 compared with diet 4, reflecting higher intake of fruits (rhamnitol, 4-hydroxyhippurate, hippurate, tartrate, and glycolate), vegetables (N-acetyl-S-(1Z)-propenyl-cysteine sulfoxide, N-acetyl-S-methyl-cysteine sulfoxide, and S-methylcysteine sulfoxide), fish (dimethylamine), and lean white meat (1-methylhistidine and 3-methylhistidine). By contrast, nine metabolites were present in higher concentrations after consumption of diet 4, which had higher amounts of red meat (O-acetylcarnitine, carnitine, and creatine) and sugars (glucose), than after consumption of diet 1. To validate the ability of their model to independently predict dietary patterns in a free-living population, the investigators used data from 24 h urine samples of 225 participants in the INTERMAP UK cohort and spot urine samples from a cohort of 66 healthy omnivorous Danish participants. In both validation studies, urinary metabolite patterns from participants with high Dietary Approaches to Stop Hypertension (DASH) scores, which are associated with reduced risk of cardiovascular diseases,^9^ clustered towards urinary metabolite profiles of diet 1, whereas urinary samples from participants with low DASH scores clustered towards urinary metabolite profiles of diet 4.

Garcia-Perez and colleagues’ study represents one of the first steps to identify objective biomarkers of dietary patterns with metabolomics. Although the preliminary results are promising, a valid dietary biomarker needs to be both sensitive and specific.^10^ In both the metabolite profiling trial and the two validation studies, differences in metabolites concentrations across the various diets were fairly modest, indicating relatively low sensitivity. The identified metabolites might not be specific to the dietary pattern of interest because they come from foods and nutrients that are likely to be shared across different dietary patterns. This issue is reflected by substantial overlap in the predicted metabolite scores across the dietary patterns. The relatively low sensitivity and specificity of dietary pattern biomarkers might also reflect the fact that the concentrations of metabolites are affected not only by dietary intake but also by absorption and metabolism of the nutrients or foods, as well as the abundance and types of gut microbiota.^11^ An additional concern is that, in the two validation studies, urinary metabolic models derived from only one urine sample (eg, 24 h or spot urine samples) cannot capture the true variation in diet and long-term dietary patterns. Finally, whether these urinary metabolites truly represent habitual dietary patterns—the aetiologically relevant exposure in nutritional epidemiology—needs to be tested by examining their relation with chronic diseases in long-term prospective studies.

Diet is a complex, multidimensional exposure, and its assessment requires a multipronged approach, depending on the objectives of the study, study populations, and study design. Although high-throughput nutritional metabolomics has offered a new and exciting tool for objective dietary assessment, it is complementary to, rather than a replacement of, traditional assessment tools such as validated dietary questionnaires and established nutrient biomarkers. To achieve the goal of precision nutrition, more efforts are needed to develop, validate, and refine assessment methods that can capture the multidimensional nature of diet.

## References

[1] Satija A, Yu E, Willett WC, Hu FB (2015). Understanding nutritional epidemiology and its role in policy. Adv Nutr.

[2] Guertin KA, Moore SC, Sampson JN (2014). Metabolomics in nutritional epidemiology: identifying metabolites associated with diet and quantifying their potential to uncover diet-disease relations in populations. Am J Clin Nutr.

[3] Edmands WM, Ferrari P, Rothwell JA (2015). Polyphenol metabolome in human urine and its association with intake of polyphenol-rich foods across European countries. Am J Clin Nutr.

[4] Mennen LI, Sapinho D, Ito H (2006). Urinary flavonoids and phenolic acids as biomarkers of intake for polyphenol-rich foods. Br J Nutr.

[5] Garg R, Brennan L, Price RK (2016). Using NMR-based metabolomics to evaluate postprandial urinary responses following consumption of minimally processed wheat bran or wheat aleurone by men and women. Nutrients.

[6] Gibbons H, McNulty BA, Nugent AP (2015). A metabolomics approach to the identification of biomarkers of sugar-sweetened beverage intake. Am J Clin Nutr.

[7] Garcia-Aloy M, Llorach R, Urpi-Sarda M (2014). Novel multimetabolite prediction of walnut consumption by a urinary biomarker model in a free-living population: the PREDIMED study. J Proteome Res.

[8] Garcia-Perez I, Posma JM, Gibson R (2017). Objective assessment of dietary patterns by use of metabolic phenotyping: a randomised, controlled, crossover trial. Lancet Diabetes Endocrinol.

[9] Salehi-Abargouei A, Maghsoudi Z, Shirani F, Azadbakht L (2013). Effects of Dietary Approaches to Stop Hypertension (DASH)-style diet on fatal or nonfatal cardiovascular diseases—incidence: a systematic review and meta-analysis on observational prospective studies. Nutrition.

[10] van Dam RM, Hunter D, Willett WC (2013). Biochemical indicators of dietary intake. Nutritional epidemiology.

[11] Sun Q, Wedick NM, Pan A (2014). Gut microbiota metabolites of dietary lignans and risk of type 2 diabetes: a prospective investigation in two cohorts of U.S. women. Diabetes Care.

